# Histozoic myxosporeans infecting the stomach wall of elopiform fishes represent a novel lineage, the Gastromyxidae

**DOI:** 10.1186/s13071-015-1140-7

**Published:** 2015-10-09

**Authors:** Mark A. Freeman, Árni Kristmundsson

**Affiliations:** Ross University School of Veterinary Medicine, Basseterre, St. Kitts West Indies; Institute of Ocean and Earth Sciences, University of Malaya, Kuala Lumpur, Malaysia; Institute for Experimental Pathology, University of Iceland, Reykjavik, Iceland

**Keywords:** *Gastromyxum*, Monomyxidae, *Monomyxum*, Elopiformes, Monogenea, Histozoic, Hyperparasite

## Abstract

**Background:**

Traditional studies on myxosporeans have used myxospore morphology as the main criterion for identification and taxonomic classification, and it remains important as the fundamental diagnostic feature used to confirm myxosporean infections in fish and other vertebrate taxa. However, its use as the primary feature in systematics has led to numerous genera becoming polyphyletic in subsequent molecular phylogenetic analyses. It is now known that other features, such as the site and type of infection, can offer a higher degree of congruence with molecular data, albeit with its own inconsistencies, than basic myxospore morphology can reliably provide.

**Methods:**

Histozoic gastrointestinal myxosporeans from two elopiform fish from Malaysia, the Pacific tarpon *Megalops cyprinoides* and the ten pounder *Elops machnata* were identified and described using morphological, histological and molecular methodologies.

**Results:**

The myxospore morphology of both species corresponds to the generally accepted *Myxidium* morphotype, but both had a single nucleus in the sporoplasm and lacked valvular striations. In phylogenetic analyses they were robustly grouped in a discrete clade basal to myxosporeans, with similar shaped myxospores, described from gill monogeneans, which are located at the base of the multivalvulid clade. New genera *Gastromyxum* and *Monomyxum* are erected to accommodate these myxosporean taxa from fish and gill monogeneans respectively. Each are placed in a new family, the Gastromyxidae with *Gastromyxum* as the type genus and Monomyxidae with *Monomyxum* as the type genus.

**Conclusions:**

To improve modern systematics of the myxosporeans it is clear that a combination of biological, ecological, morphological and molecular data should be used in descriptive studies, and the naming and redistribution of taxa and genera is going to be necessary to achieve this. Here we demonstrate why some *Myxidium*-shaped myxospores should not be included in the family Myxidiidae, and create two new families to accommodate them based on their site of infection, host biology / ecology, DNA sequence data and morphological observations. Subsequent descriptive works need to follow a similar course if we are going to create a prevailing and workable systematic structure for the Myxosporea.

## Background

Myxosporeans are common parasites of fish and in recent molecular taxonomic studies they form two main groups, being mainly from hosts that inhabit either freshwater or marine environments [1, 2]. Known myxosporean life cycles involve two hosts, with infections in fish resulting in the production of myxospores, whilst morphologically dissimilar actinospores are produced in an annelid invertebrate host. Traditional studies on myxosporeans have used myxospore morphology as the main criterion for identification and taxonomic classification. This has led to numerous genera becoming polyphyletic in subsequent molecular phylogenetic analyses and it is now known that other features, such as the site of infection in fish, can, for some clades of myxosporeans, offer a far higher degree of congruence with molecular data, than basic myxospore morphology can provide [1, 3–5]. However, myxospore morphology remains important, as it is the fundamental diagnostic feature that veterinarians and scientists use for identification and it remains the distinguishing characteristic of myxosporean infections in fish and other vertebrate taxa.

*Myxidium incomptavermi* was described from *Myxidium*-shaped myxospores found infecting monogeneans from the gills of *Megalops cyprinoides* in Malaysia [6]. However, monogeneans are not typical hosts for myxosporeans and myxospores are not normally found in the invertebrate host. In that study, no myxospores of *M. incomptavermi* were found in the fish that were host to the infected monogeneans and multiple fish were found to have monogeneans that were infected with myxospores. But, a specific PCR was able to detect the DNA of *M. incomptavermi* in numerous tissues of the fish host, in particular the stomach and intestine, suggesting that the fish might be involved in the life cycle or the transmission of *M. incomptavermi* to gill monogeneans [6].

The aim of the present study was to screen for the presence of gastrointestinal myxosporeans from two elopiform fishes (order: Elopiformes), from Malaysian mangrove systems, the Pacific tarpon *M. cyprinoides* and the ten pounder *Elops machnata*. Both fish species are found in shallow coastal marine and brackish water environments, entering the mangroves during the night. The ten pounders or ladyfish (Elopiformes: Elopidae), and tarpons (Elopiformes: Megalopidae) together with their sister group the eels (Anguilliformes) form the Elopomorpha which are one of the oldest major extant teleost lineages [7, 8].

## Methods

*Elops machnata* were purchased from the main fish market in Kuah, Langkawi. *Megalops cyprinoides*, were bought from the same market and also captured whilst night fishing with gill nets in Kilim mangroves, northeast Langkawi, and kept alive prior to examination. In addition, *M. cyprinoides* were purchased from the fish market at Pangkor Island, Perak and captured live at the Bachok Marine Research Station, Kelantan. All fish were examined for the presence of histozoic myxosporeans in the gastrointestinal tract by scraping the stomach and intestine lining with a scalpel blade and viewing with an Olympus BX-41 compound microscope. Images of fresh myxospores were taken using a Leica DMLB digital camera using Cell imaging software. The dimensions of at least 20 myxospores from each host fish were calculated using Image J 1.42q [9] following the accepted guidelines of Lom & Arthur [10]. *Elops machnata* were also examined for the presence of gill monogeneans, by removing each gill arch, placing it in seawater and observing between the primary filaments using a dissecting microscope.

Tissue samples that were shown to be positive for myxosporeans were fixed in 10 % buffered formalin for histological analysis. After fixation, tissues were prepared for standard wax histology and 4 μm sections were dewaxed, stained with Giemsa and examined using a compound microscope. Positive tissue samples were also fixed for transmission electron microscopy (TEM) as previously described [11]. Fish that were found to have heavy infections were used to prepare samples for scanning electron microscopy (SEM). For the SEM examination, myxospores were removed from heavily infected stomach wall tissues by scraping with a scalpel and placing in a tube containing PBS. Tubes were then centrifuged at 1500g for 5 min, the supernatant removed and the pellet fixed in 2.5 % glutaraldehyde for 4h. After fixation, the spores were prepared for SEM and viewed following the methods described by Kristmundsson and Freeman [12]. In brief, washed spores were syringed onto a polycarbonate membrane, fixed with 1 % osmium tetroxide and dehydrated through an ethanol series. Membranes were dried, mounted on stubs, sputter-coated with gold and viewed, between 5 and 10 kV, with a FEI Quanta 450 FEG FE-SEM.

Infected gastrointestinal tissue or myxospore-positive intestinal scrapings were fixed in 95 % ethanol or placed directly into DNA lysis buffer for molecular analyses. Total DNA was extracted using a GeneMATRIX DNA isolation kit (EURx Poland) following the tissue protocol and used as templates in subsequent PCR reactions. Parasite small subunit ribosomal DNA (SSU rDNA) was amplified using the general myxosporean primers and methodology described by Freeman *et al.* [4] and the *Kudoa*-specific primer Kud-80f actgcgaagcgctcagta [13] with the new reverse primer Kud-790r cgcctgctttgagcactgtg, utilising the same PCR conditions. PCRs were conducted on parasite DNA from 4 fish for each species. A specific PCR was designed and optimised for the new myxosporean from *M. cyprinoides*, in order to be able to differentiate it from *Myxidium incomptavermi* in fish with dual infections or those with both DNA present. The primers Mi2-470f taccggagttgaccttcacg and Mi2-1040r actgatcccgtggtggcat amplified a 570bp region of SSU rDNA of this novel myxosporean, using the same PCR conditions except utilising an annealing temperature of 65C. Two additional forward primers were designed to match the new myxosporeans described in this study but not to anneal to *M. incomptavermi*. Gastro-250f actgtgcatatcgaatgggct and Gastro-1100fagtacggtcgcaaggctga were used with the general reverse primers (1430r and 18gM respectively) and PCR conditions described by Freeman *et al.* [4]. All DNA samples taken from *M. cyprinoides* were screened for *M. incomptavermi* using the specific PCR described by Freeman & Shinn [6] and spores measurements were only taken from PCR negative fish and those shown to be positive with the specific PCR (Mi2-470f / Mi2-1040r).

All PCRs were performed in triplicate and PCR products of the expected sizes were recovered using a GeneMATRIX PCR products extraction kit (EURx Poland). Sequencing reactions were performed using BigDyeTM Terminator cycle sequencing chemistry utilising the same oligonucleotide primers that were used for the original PCRs. DNA sequencing was performed in both forward and reverse directions for all PCR products and nucleotide BLAST searches performed for each sequence read to confirm a myxosporean origin [14]. The contiguous sequences were obtained manually using CLUSTAL X and BioEdit [15, 16]. CLUSTAL X was used for the initial SSU rDNA sequence alignments of the novel sequences and 31 other histozoic marine myxosporeans. Percentage divergence matrices were constructed from selected aligned taxa in CLUSTAL X using the neighbour-joining method based on the Kimura 2-parameter model [17].

Phylogenetic analyses were performed using the maximum likelihood methodology in PhyML [18] with the general time-reversible substitution model selected and 1000 bootstrap repeats, and Bayesian inference (BI) analysis using MrBayes v. 3.2 [19]. For the BI analysis, models of nucleotide substitution were first evaluated for the alignment using MrModeltest v. 2.2 [20]. The most parameter-rich evolutionary model based on the AIC was the general time-reversible, GTR + I + G model of evolution. Therefore, the settings used for the analysis were nst = 6, with the gamma-distributed rate variation across sites and a proportion of invariable sites (rates = invgamma). The priors on state frequency were left at the default setting (Prsetstatefreqpr = dirichlet (1,1,1,1)). Posterior probability distributions were generated using the Markov Chain Monte Carlo (MCMC) method with four chains being run simultaneously for 1,000,000 generations. Burn in was set at 2500 and trees were sampled every 100 generations making a total of 7500 trees used to compile the majority rule consensus trees.

## Results and discussion

Six *Elops machnata* ranging in size from 39–81 cm in fork length (FL) and a total of 38 *Megalops cyprinoides*, 26 from Langkawi (FL 19–32 cm), 9 from Pangkor (FL 17–30) and 3 from Bachok (FL 20–25) were dissected and checked for the presence on myxospores under the microscope. All six *E. machnata* (100 % prevalence) had a histozoic myxosporean infecting the stomach wall. A total of 17 *M. cyprinoides* (45 % prevalence) also had a myxosporean in the stomach wall, 10 from Langkawi (38 % prevalence), seven from Pangkor (78 % prevalence) and none from Bachok (0 % prevalence). No morphologically different myxosporeans were found lower in the intestinal tract in either fish species and spore density was highest from the stomach tissue. No monogeneans were found on the gills of *E. machnata*, the gills of *M. cyprinoides* were not examined in this study.

**Fig. 1:**
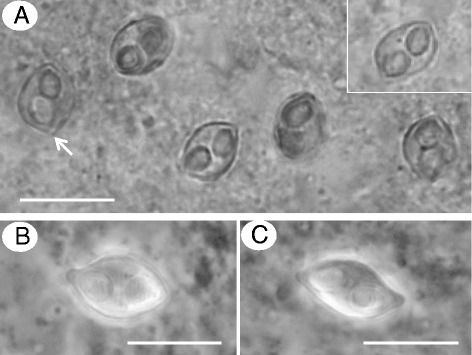
Light micrographs of *Gastromyxum rafii* n. sp. and *Gastromyxum bulani* n. sp. **a** Myxospores of *G. rafii* are bluntly rounded and rhomboid in appearance when in the sutural plane (inset), but have raised tips where the polar capsules discharge (arrow). Myxospores of *G. bulani* are lemon-shaped in the valvular view (**b**) and moderately sigmoidal in the sutural view with prominent nipple-like structures at each apex (**c**). Scale bars 10 μm

**Fig. 2:**
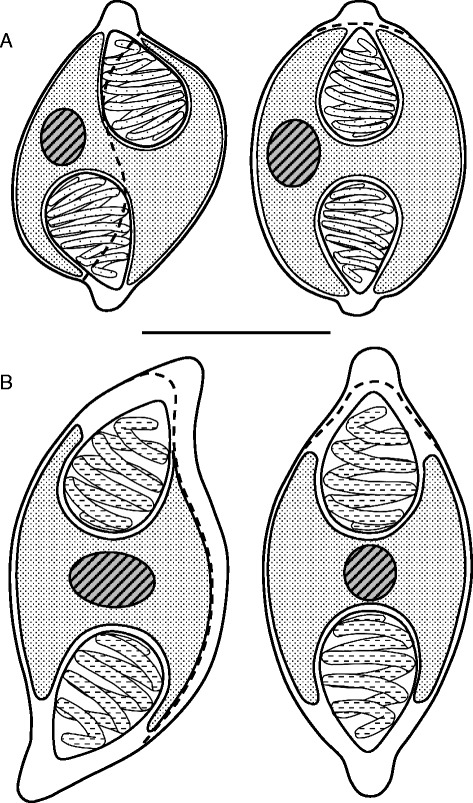
Line drawings of myxospores of (**a**) *Gastromyxum rafii* n. sp. and (**b**) *Gastromyxum bulani* n. sp. Spores in sutural view on left hand side, the position of the suture is shown with a dashed line. Spores shown in valvular view on the right hand side. Scale bar 5 μm

In *E. machnata* the myxospores were somewhat reminiscent to that of *Myxidium* or *Zschokkella* in basic morphology but had more rounded ends with raised tips where the polar capsules discharge and a clear rhomboid shape in the sutural view (Figs. 1a and 2a). Polar capsules were large and pyriform with 6 coils of the polar filament and, depending on the angle of view, appeared to almost meet in the centre of the spore, with the single nucleus to one side (Figs. 1a and 2a). Fresh spores measured 7.92 (7.1–8.5) μm in length and 5.42 (4.9–5.9) μm in width, polar capsules measured 3.3 (3.0–3.5) μm in length and 2.36 (2.1–2.5) μmin width *n* = 60 (3 fish). In *M. cyprinoides* the myxospores had a more *Myxidium*-like morphology, being moderately sigmoidal in the sutural view but lemon-shaped in the valvular view (Figs. 1b,c and 2b). Polar capsules were pyriform with 4 coils of the polar filament with a single centrally positioned nucleus between them (Fig. 2b). Fresh spores measured 10.29 (7.84–11.56) μm in length and 5.64 (4.72–6.78) μm in width, polar capsules measured 3.40 (2.63–4.32) μm in length and 2.75 (1.69–3.87) μm in width *n* = 138 (5 fish).

**Fig. 3:**
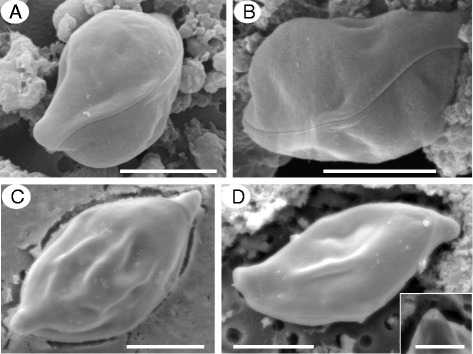
Scanning electron micrographs of *Gastromyxum rafii* n. sp. and *Gastromyxum bulani* n. sp. *Gastromyxum rafii* spores have rounded ends (**a**) and are rhomboid in the sutural view (**b**). *Gastromyxum bulani* spores are lightly fusiform in the valvular view (**c**), sigmoid in the sutural view (**d**) and have more pointed ends with an apical opening for the polar filament to be released through (inset **d**). Both species have smooth valves and non-prominent sutural lines. Each valve has one rounded/pointed end which forms a nipple-like structure at its apex, with the other end concealed beneath the apex of the opposing valve. Scale bars 3 μm (inset 1 μm).

Scanning electron micrographs revealed that both spore types had smooth valves with a sinuous and relatively inconspicuous sutural line, each valve possessed one apical element, through which the polar filament discharges, with the other end tucking under the valve edge of the apical element of the opposing valve (Fig. 3a-d).

**Fig. 4:**
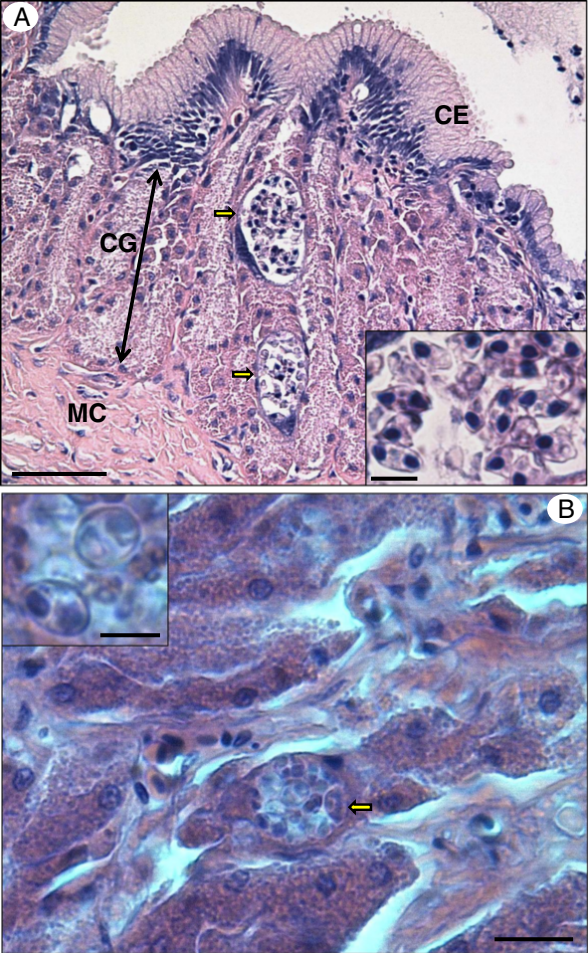
Giemsa-stained histological sections of *Gastromyxum rafii* n. sp. and *Gastromyxum bulani* n. sp. **a** Histological section of the stomach of *M. cyprinoides* detailing the structures: CE columnar epithelium, CG cardiac glands, MC muscle coat. Two polysporous myxosporean plasmodia are present in the cardiac gland tissue (arrows) each containing numerous myxospores (inset). Bar = 50μm and 10μm inset. **b** Histological section from *E. machnata* showing a polysporous plasmodia (arrow) in the glandular tissue of the stomach wall, containing mature myxospores (inset). Bar = 25μm and 10μm inset

Histological examination showed that myxosporean infections in both species of fish were histozoic in the glandular part of the stomach wall, with myxospores developing in polysporous plasmodia with no sign of significant pathology or host responses being evident (Fig. 4a and b).

**Fig. 5:**
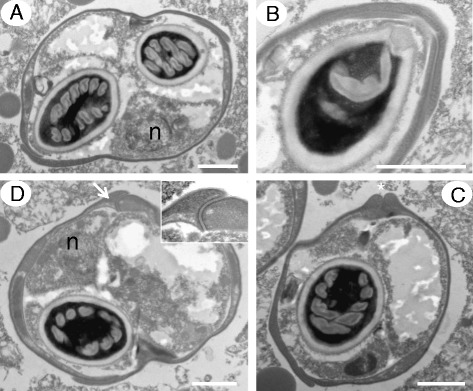
Transmission electron micrographs of *Gastromyxum rafii* n. sp. Spores have a single nucleus (n) and valves that interlock at the apical ends with a substantial overlap (**a**). The overlapping valves form a complex double layer, around parts of the polar capsule (**b**), which is not present at the apex of the polar capsule where the polar filament is discharged (asterisk) (**c**). Away from the apical ends of the spore, the vales meet to form a hinge-like structure (arrow) (**d** and inset). Scale bars 1 μm.

**Fig. 6:**
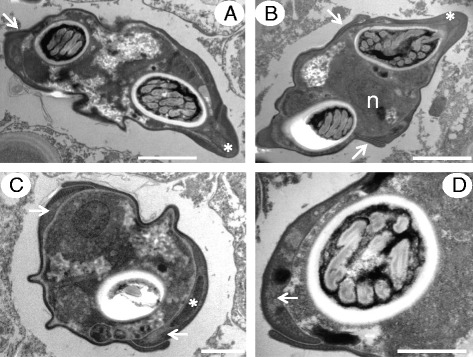
Transmission electron micrographs of *Gastromyxum bulani* n. sp. Spores have a single nucleus (n) and valves that join with an overlap (arrows) and are considerably thickened at the apical end (asterisk), away from the suture where the polar filament is discharged (**a** and **b**). At the margin of one end of each valve are the valvogenic cells (arrows) and nuclei (asterisk) (**c** and **d**). Scale bars 2 μm (**a**,**b**) 1 μm (**c**,**d**)

Transmission electron microscopy of mature myxospores of both novel species reveal interlocking valves with a substantial overlap in places that forms a non-prominent almost flush sutural line. Similar ultrastructural observations were also made during the description of the genus *Enteromyxum* [21]. The valves are heavily thickened in areas leading to the tip of the spore where the polar filament is discharged and sometimes meet to form a hinge-like structure, again with a flush suture (Figs. 5 and 6).

Almost complete SSU rDNA sequences were obtained for both myxosporeans using the aforementioned PCRs and assigned the GenBank accession numbers KT002405/6, with 1745 bp sequenced for the myxosporean from *E. machnata* and 1662 bp sequenced for the myxosporean from *M. cyprinoides*. BLAST searches of the contiguous sequences showed that the closest relative in the databases was *Myxidium incomptavermi* for both myxosporeans, with similarities of 91 %. The PCR using the primer pair Mi2-470f / Mi2-1040r only amplified the myxosporean from *M. cyprinoides* and did not amplify DNA from *M. incomptavermi*. Likewise, both new forward primers Gastro-250f and Gastro-1100f, when used with universal myxosporean reverse primers, only amplified the two novel myxosporeans and did not amplify *M. incomptavermi*. DNA samples from the stomach wall of some *M. cyprinoides* (5 fish) tested weakly positive for *M. incomptavermi*, therefore these fish were not used for myxospore evaluation; however, no morphologically dissimilar myxospores were observed from these five fish.

Due to the distinct nature of these two myxosporeans with respect to host, site of infection and DNA sequence data, new species descriptions are made and we propose to place the two novel myxosporeans from this study in a new genus *Gastromyxum *n. gen. (*Gastromyxum bulani* n. sp. from *M. cyprinoides* and *Gastromyxum rafii* n. sp. from *E. machnata*). In addition, we suggest transferring the two species that infect gill monogeneans, *Myxidium incomptavermi* [6] and the undescribed *Myxidium* sp. from Japan (GQ368245) to a new genus *Monomyxum* n. gen. (*Monomyxum incomptavermi* n. comb. and *Monomyxum* sp.).

A percentage identity matrix of these myxosporeans and phylogenetically related taxa (Table 1) shows that the novel species from the present study, *G. bulani* and *G. rafii*, from stomach wall infections in marine fishes are most similar to each other with a 94.8 % identity. These species are less similar to both *Monomyxum incomptavermi* and *Monomyxum* sp. from gill monogeneans with 89–91 % identity. However, similarity between the two *Monomyxum* spp. from gill monogeneans is 93.1 %. There is a further reduction in similarity with other myxosporean taxa to *Gastromyxum* spp., 87.2–87.7 % to *Kudoa* spp., 86.3–86.4 % to *Unicapsula* sp., 83–83.8 % to *Enteromyxum leei* and 77.2–77.9 % to the type species of *Myxidium* (*M. lieberkuehni*).

**Table 1:** Percentage identities of SSU rDNA sequences, above diagonal, and number of bases compared, below diagonal, for *Gastromyxum* spp. and phylogenetically related myxosporeans and the type species of *Myxidium*, *M. lieberkuehni*

	1	2	3	4	5	6	7	8	9
(1) *Gastromyxum elopsi*	-	94.8	90.9	89.4	87.7	87.2	86.4	83.8	77.9
(2) *Gastromyxum bulani*	1657	-	90.8	88.9	87.3	87.6	86.3	83.0	77.2
(3) *Monomyxum incomptavermi*	1695	1635	-	93.1	89.6	89.1	88.8	84.1	77.3
(4) *Monomyxum* sp.	1694	1634	1696	-	88.1	87.7	87.9	83.0	76.1
(5) *Kudoa thyrsites*	1635	1576	1630	1633	-	91.2	88.4	81.9	75.0
(6) *K. amamiensis*	1559	1498	1556	1558	1558	-	88.4	82.0	74.2
(7) *Unicapsula* sp.	1657	1598	1652	1655	1635	1572	-	83.0	76.7
(8) *Enteromyxum leei*	1571	1490	1547	1548	1543	1522	1540	-	77.0
(9) *Myxidium lieberkuehni*	1632	1551	1604	1606	1604	1586	1616	1573	-

Although the morphology of *Gastromyxum* and *Monomyxum* myxospores share similarities to each other and to the generally accepted *Myxidium* morphotype, we propose to create two new families to accommodate these new genera. The Gastromyxidae n. fam., type genus *Gastromyxum*, and the Monomyxidae n. fam., type genus *Monomyxum*. These new families both represent distinct marine myxosporean lineages, each with unique features that differ from *Myxidium sensu stricto* in host type, spore morphology, tissue location and development and DNA sequence data. The type species of *Myxidium*, *M. lieberkuehni* is commonly found as coelozoic in the urinary system of the freshwater pike, *Esox lucius*, in the holarctic region of the northern hemisphere, it has elongated and striated myxospores with a sporoplasm containing two nuclei unlike those in the current description that have a single nucleus and smooth spore valves. In addition, *M. lieberkuehni* is robustly phylogenetically placed away from marine myxosporeans in a discrete clade with other coelozoic species infecting freshwater fishes [1, 12, 22].

**Fig. 7:**
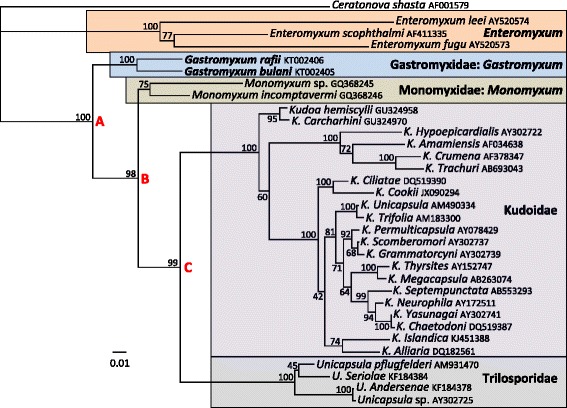
Maximum likelihood topology based on 1851 characters of aligned small subunit rDNA of 33 histozoic marine myxosporeans. The phylogenetic tree has a well-supported spine (nodes A-C), forming three distinct and robustly supported groups. *Gastromyxum rafii* and *G. bulani* form a fully supported clade from node A which represents the new family and genus Gastromyxidae: *Gastromyxum* (blue area). The two species known from monogeneans are well-supported from node B, and represent the new family and genus Monomyxidae: *Monomyxum* (brown area). From node C, the Kudoidae (purple area) and Trilosporidae (grey area), the two families that comprise the multivalvulida, form robustly supported sister clades. Basal to the Gastromyxidae is the well-supported clade of intestinal myxosporeans comprising the *Enteromyxum* spp. (orange area), whilst the histozoic intestinal myxosporean *Ceratonova shasta* was used as an outgroup. Values at the nodes represent bootstrap support from 1000 resamplings

Phylogenetic analyses of some of the marine histozoic myxosporeans consistently and robustly place members of the Gastromyxidae and Monomyxidae at the base of the Multivalvulida (Kudoidae and Trilosporidae) (Fig. 7). With the currently available DNA data, members of the *Enteromyxum*, *Gastromyxum* and *Monomyxum* genera form separate but discrete and well-supported clades in this histozoic group (Fig. 7). The two *Monomyxum* spp. have not always convincingly formed a discrete clade, and in the analyses by Freeman and Shinn [6] they were unresolved but adjacent taxa in the tree. However, the addition of the two *Gastromyxum* sequences has added stability to this part of the phylogenetic tree. To confirm this we aligned the two novel *Gastromyxum* sequences to the original alignment file used in the Freeman and Shinn publication [6]. With the *Gastromyxum* sequences added to the original alignment, a maximum likelihood analysis (performed as previously described [6]) also placed the two *Monomyxum* sequences in a discrete clade with a support of 82 % (data not shown). This confirms the phylogenetic relationship, within *Monomyxum*, that we demonstrate in Fig. 7, which supports the creation of the novel family Monomyxidae for *Monomyxum* spp. In addition, in other recent extensive phylogenetic studies of the myxosporeans, even without the inclusion of the *Gastromyxum* sequences, it has also been shown that the two *Monomyxum* sequences are placed together in a discrete clade, albeit only with moderate support [23]. In the same study, it was demonstrated that *Ceratonova* spp. are related to the *Enteromyxum* and *Kudoa* clades [23], and their relationship to *Enteromyxum* and the Gastromyxidae is further supported as they are also histozoic parasites in the intestine of fishes. However, the genus *Ceratonova* was recently placed in the family Ceratomyxidae [24]. This reversion to myxospore morphology as the primary taxonomic criterion could be due to the complications of the freshwater hosts for *Ceratonova* spp., and the early inclusion of *Ceratonova shasta* in *Ceratomyxa*, but is more likely due to the lack of a suitable family placement for these enteric histozoic ceratomyxid forms. As the region of the phylogenetic tree for enteric myxosporeans is relatively sparse, we are unable to expand the boundaries of the Gastromyxidae to include other genera and create a pararphyletic family. However, the placement of *Enteromyxum* spp. in the Myxidiidae and *Ceratonova* spp. in the Ceratomyxidae is incorrect, and the erection of new families to accommodate these genera is warranted in the future.

### Taxonomic summary

Class Myxosporea Bütschli, 1881

Order Bivalvulida Shulman, 1959

Suborder Variisporina Lom et Noble, 1984

Gastromyxidae n. fam. Freeman et Kristmundsson, 2015

Spores are generally fusiform, being sigmoid to rhomboid in the sutural view and lemon to crescent-shaped and in valvular view, with no striations and an inconspicuous curved sutural line that bisects the spore but not through the pointed extremities associated with the polar capsule apex. Two large tear-shaped to pyriform polar capsules lie at opposite ends of the spore, with a single nucleus between them. Polar filaments are discharged, in opposing directions, through capsular foramina that are located at the rounded or pointed extremities of the spore valves. Histozoic in the gastrointestinal tract of marine fishes forming polysporous plasmodia.

Single genus: *Gastromyxum *n. gen. (type genus)

*Gastromyxum* n. gen.

With characteristics of the family. Spores are sigmoid to rhomboid in the sutural view and lemon-shaped in valvular view. Polar capsules are large and tear-shaped to pyriform. Histozoic in the stomach of marine fishes, forming polysporous plasmodia in the glandular part of the stomach wall. Not pathogenic to fish with little or no host tissue responses observed. Two species, possibly three see *Myxidium elopsi*.

Etymology : Gastro from the Greek gastros meaning stomach, refers to the tissue location.

*Gastromyxum bulani* n. sp. (type species)

Type locality: Coastal waters of Langkawi Island, Peninsular Malaysia

Type host: *Megalops cyprinoides* (Broussonet, 1782)

Site of infection: cardiac glands of stomach wall

Etymology : *bulani* refers to the Malay name for the fish host, ikan bulan

Type material: two Giemsa stained histological slides, a Diff Quik stained slide of fresh spores and frozen DNA sample have been deposited at the Institute for Experimental Pathology at Keldur, University of Iceland, with the following registration numbers (2015.110 -14).

*Gastromyxum rafii* n. sp.

Type locality: Coastal waters of Langkawi Island, Peninsular Malaysia

Type host: *Elops machnata* (Forsskål, 1775)

Site of infection: Cardiac glands of stomach wall

Etymology : *rafii* refers to the name of a friend from Langkawi that sadly recently passed away.

Type material: Two Giemsa stained histological slides, a DiffQuik stained slide of fresh spores and frozen DNA sample have been deposited at the Institute for Experimental Pathology at Keldur, University of Iceland, with the following registration numbers (2015.120 -24).

Monomyxidae n. fam. Freeman et Kristmundsson, 2015

Spores are fusiform, being sigmoid in the sutural view and lemon-shaped and in valvular view, with no striations and an inconspicuous curved sutural line that bisects the spore but not through the pointed extremities associated with the polar capsule apex. Two large tear-shaped to pyriform polar capsules lie at opposite ends of the spore, with a single nucleus between them. Polar filaments are discharged, in opposing directions, through capsular foramina that are located at the pointed ends of the spore valves. Histozoic in the parenchymal tissues of gill monogeneans probably forming disporous pseudoplasmodia (not confirmed).

Single genus: *Monomyxum* n. gen. (type genus)

*Monomyxum* n. gen.

Spores are fusiform and sigmoid in the sutural view and lemon-shaped in valvular view. Polar capsules are large and tear-shaped to pyriform. Histozoic in the parenchymal tissues of gill monogeneans from marine fish, may form disporous plasmodia (not confirmed). Infected monogeneans appear to be moribund or senescent indicating pathogenicity. Two species.

Etymology : mono refers to the monogenean host.

*Monomyxum incomptavermi* n. comb. (type species) (syn *Myxidium incomptavermi*) Freeman et Shinn, 2011

Site of infection: Parenchymal tissues of monogenean host. Described from myxospores infecting the gill monogenean *Diplectanocotyla gracilis* from *M. cyprinoides* caught from the coastal waters of Langkawi Island, Peninsular Malaysia.

Also detected by PCR in certain tissues (kidney, spleen, intestinal tract) from the fish host, *M. cyprinoides*, but no myxospores yet encountered in fish host. Myxospore morphology is very similar to that of *Gastromyxum bulani*, the latter being identified using diagnostic PCRs to identify hosts with no trace amplifications of *M. incomptavermi*.

*Monomyxum* sp. (see Freeman *et al.* [25])

Site of infection: Parenchymal tissues of monogenean host. Reported from gill monogenea, *Haliotrema* sp. from a *Platycephals s*p. collected from Lake Hamana, Japan. Resampling to complete a full species description has been unsuccessful.

Zoobank information: This manuscript has been registered with ZooBank under the following lsid.:zoobank.org:pub:A888924F-8EC6-48C3-BA0E-D9798963BF5E

Nomenclature acts:

Gastromyxidae n. fam.: lsid:zoobank.org:act:E6D87E29-74AC-4138-9355-B11D383C13E6

*Gastromyxum* n. g.: lsid:zoobank.org:act:BA1B4338-60C8-425F-8194-4EF024724F12

*Gastromyxum bulani* n. sp.: lsid:zoobank.org:act:994E49B5-AE7A-4A7C-B442-A00DA01EFD70

*Gastromyxum rafii* n. sp.: lsid:zoobank.org:act:96457F71-D0F3-4FE5-80AE-0BE2E76C9771

Monomyxidae n. fam. : lsid:zoobank.org:act:C1417121-3534-44E7-A69A-BC4AECC37269

*Monomyxum* n. g.: lsid:zoobank.org:act:783DAEA0-DE07-4270-918D-1ACF95049206

*Monomyxum incomptavermi* n. comb.: lsid:zoobank.org:act:7DD6B3CC-8124-41D1-99C8-C0A6248BFF02

It is not known whether *M. incomptavermi* infections develop to form myxospores in the fish host and no myxospores were detected from fish in earlier studies [6]. However, the stomach wall was not thoroughly examined in that study, assuming it might potentially be the site of infection for *M. incomptavermi* in fish, as it is for *G. bulani*. In the present study, myxospores of *G. bulani* and *G. rafii* were deeply embedded in the glandular tissue of the stomach which had to be scraped sufficiently hard to release spores for identification in fresh preparations using a compound microscope. It is possible that other infections, in the glandular part of the stomach, have gone unnoticed due to this reason and that this tissue is a more common site for myxosporean infections in fish than is currently known. Spores of *M. incomptavermi* measured from gill monogeneans infecting *M. cyprinoides* [6] were very similar in shape to those of *G. bulani* but were more fusiform and slender in form, being slightly longer, 11.62 μm (11.27–11.75) and less broad 4.92 μm (4.19–5.56). In the present study, no myxospores conforming to the shape of *M. incomptavermi* were encountered; however, DNA from *M. incomptavermi* was detected using a specific PCR from 5 fish indicating its presence in some form. Therefore, it still remains unknown if *M. incomptavermi* infections develop in fish or whether they are just transient or dead-end infections, but the prevalence of *G. bulani* is much higher and readily detected as myxospores from the stomach wall. It is possible, even likely, that *M. incomptavermi* and *G. bulani* share an unknown common ancestor as they have very similar spore morphologies, are detected from the same fish host and have nearly 90 % similarity with respect to SSU rDNA sequences. However, more sequence data from additional taxa are required to fully clarify the inter-family relationships for this part of the myxosporean tree. Indeed, *M. incomptavermi* has a higher similarity in the SSU rDNA (93.1 %) to *Monomyxum* sp. from a monogenean from Japan found on an unrelated fish (*Platycephalus* sp.) and *G. bulani* and *G. rafii* also share a greater similarity in the SSU rDNA (94.8 %) despite being in different fish hosts and having more significant differences in myxospore morphology (Figs. 1, 2, 3). These generic relationships are confirmed in the phylogenetic analyses (Fig. 7) which support monogeneans as being true hosts for myxosporeans and demonstrates that the two *Monomyxum* spp. share a more recent common ancestor than they do to either *Gastromyxum* sp. from fish and vica versa. However, this allows us to speculate that myxospore morphology may be more conserved in certain host systems. *Gastromyxum bulani* and *M. incomptavermi* share very similar myxospore morphologies and are both found on the same species of fish, albeit one in a monogenean; however, they are less genetically related to each other than each is to another, different shaped, myxospore form in a different fish / monogenean host system. This might suggest that evolutionary changes in myxospore morphology could occur at a slower rate in one species of fish, even when becoming hyperparsitic in their gill monogeneans, than it does when a switching of fish host has occurred, even if the fish are found in the same environment, as is the case for *M. cyprinoides* and *E. machnata*. Put simply, if myxospore morphology evolved evenly in all host systems, we might expect the more closely related *Gastromyxum* spp. to look more similar, when in fact, *G. bulani* looks remarkably similar to *M. incomptavermi*, known from the same fish host, despite being more distantly related to it. It is also possible that it is a coincidence that these spores have near identical morphologies, however, we consider this unlikely as morphological variability among *Myxidium-*shaped spores is wide-ranging [26], and the similarities between these two species is very striking. It is also noteworthy that *G. bulani* and *G. rafii* both infect elopiform fishes (order: Elopiformes), from mangrove systems from the west coast of peninsular Malaysia, suggesting that elopiform fish may be a more common host to *Gastromyxum* spp.. Interestingly, the myxosporean, *Myxidium elopsi*, described from the intestine of *Elops senegalensis* from the Atlantic coast of West Africa [27] has a very similar spore morphology to *G. rafii* and is found as histozoic in the intestine of a closely related elopiform fish. It is highly likely that *M. elopsi* belongs in the genus *Gastromyxum*, and its similar shape to *G. rafii* and elopiform host further support the theories that myxospore morphology is potentially better conserved in a single species or closely related fish species (same genus), and that elopiform fishes may be a common host for *Gatromyxum* spp.

No monogeneans were found on the gills of *E. machnata* in this study and PCR testing of gill monogenean DNA from *M. cyprinoides* from a previous study [6], using specific PCRs designed in this study for *Gastromyxum*, did not amplify *G. bulani*. This suggests that *Gastromyxum* spp. do not infect gill monogeneans, but this will require further research effort to unambiguously demonstrate.

Traditional myxosporean taxonomy has been heavily dependent upon the morphology of myxospores to differentiate between the numerous different taxa. More recently, multiple molecular phylogenetic studies of the Myxosporea have repeatedly and robustly demonstrated that the generic assignment of taxa based on myxospore morphology has resulted in a polyphyletic distribution of many genera, which has led to some genera such as *Myxidium* and *Zschokkella* becoming misleadingly high in species number, many of which are incorrectly assigned. It is also clear that some myxospore morphotypes have formed or reformed on more than one occasion during myxosporean evolution, which is probably due to comparable biological and environmental constraints and pressures that needed to be satisfied during their evolution, but does not necessarily mean they belong in the same family or genus. Many older studies provide excellent descriptions, but assign primary weight to myxospore morphology (no DNA data available) which unknowingly led to incorrect taxonomic placements with respect to genus or family. However, surprisingly numerous recent descriptions, also provide good taxonomic data but still assign primary weight to myxospore morphology, leading to generic assignments that are inconsistent with known molecular data [6, 24].

As myxospores remain our definitive confirmation of myxosporean infections in vertebrates, we must find alternative ways in which to develop a more meaningful systematic approach for the nomenclature of myxosporean taxa. As myxospore presence and morphology is often our first point of reference it must remain part of the overall approach, but must be combined with, but come second, to other potentially more meaningful data. However, it is not clear how this can be easily achieved and readily applied across the whole group. The site of infection in the host (muscle, GB, urinary system etc.) and whether development is histozoic or coelozoic is useful and gives strong congruence with molecular data for some clades but it is not clear in all, for example the parvicapsulids. Histopathology can reveal different developmental stages and important interactions with host cells, but if cross-referenced to infections at a differing stage could be misleading and may vary with both parasite and host strains. Host species and environmental data (marine, freshwater, geographic location etc.) can show good compatibility to molecular phylogenies, but again numerous exceptions exist, such as the marine myxobolids.

In short, if we want a new taxonomic framework for the myxosporeans to reflect molecular phylogenies, it is imperative to provide good molecular data with every description. But, this must be augmented with as much other relevant data as possible.

## Conclusions

During this study, it became clear that the discovery of myxosporean parasites, found as histozoic infections in the stomach of elopiform fishes from Malaysia, were novel and that they required taxonomic description. However, when we evaluated all the taxonomic information gathered herein, no family placement or generic assignment was suitable. Therefore, we have placed less emphasis on myxospore morphology and created a new family and genus, the Gastromyxidae: *Gastromyxum*, to accommodate them. During this process, it also became clear that myxosporean taxa infecting gill monogeneans were a distinct lineage, that have been incorrectly assigned to *Myxidium* due to the dogma of following myxospore morphology as the primary taxonomic criterion [6], and that a new generic and family assignment, the Monomyxidae: *Monomyxum*, was called for. In many cases, myxospore morphology will be able to provide useful characters within certain groups/clades, which may correspond to certain myxosporean genera, such as in the *Gastromyxum*; however, it must be used carefully and in combination with other important features for that particular group or clade of myxosporeans.

In order to resolve the perennial taxonomic issues surrounding this group, deeper taxonomic assignment and resolution is going to be required. Here we demonstrate and give sound reasons why some species with *Myxidium*-shaped myxospores should not be included in the family Myxidiidae, and subsequent descriptive works will hopefully follow a similar course so we can collectively create a modern and workable phylogenetic framework for the myxosporeans.
